# Factors that influence advance directives completion amongst terminally ill patients at a tertiary hospital in Kenya

**DOI:** 10.1186/s12904-017-0186-z

**Published:** 2017-01-25

**Authors:** Stephen Omondi, John Weru, Asim Jamal Shaikh, Gerald Yonga

**Affiliations:** Aga Khan University-Kenya, Nairobi, Kenya

**Keywords:** Advance directives, Palliative care, Terminal illness, Factors, Kenya

## Abstract

**Background:**

An advance directive (AD) is a written or verbal document that legally stipulates a person’s health care preference while they are competent to make decisions for themselves and is used to guide decisions on life-sustaining treatment in the event that they become incapacitated. AD can take the form of a living will, a limitation of care document, a do-not-resuscitate order, or an appointment of a surrogate by durable power of attorney. The completion rate of AD varies from region to region, and it is influenced by multiple factors. The objectives of this study were to determine the proportion of terminally ill patients with AD and to identify the factors that influence the completion of AD amongst terminally ill patients at a tertiary hospital in Kenya.

**Methods:**

The study was a retrospective survey. All available records of terminally ill patients seen at Aga Khan University Hospital, Nairobi, between July 2010 and December 2015, and that met the inclusion criteria were included in the study.

**Results:**

In total, 216 records of terminally ill patients were analyzed: 89 records were of patients that had AD and 127 records were of patients that did not have AD. The proportion of terminally ill patients that had completed AD was 41.2%. The factors that were associated with the completion of AD on bivariate analysis were history of ICU admission, history of endotracheal intubation, functional status of the patient, the medical specialty taking care of the patient, patient’s caregiver discussing the AD with the patient, and a palliative specialist review. On multivariate regression analysis, discussion of AD with a caregiver and patient’s functional impairment were the factors with statistically significant association with completion of AD.

**Conclusions:**

The proportion of terminally ill patients that had AD in their medical records was significant. However, most terminally ill patients did not have AD. Our data, perhaps the first on the subject in East Africa, suggest that most of the factors associated with AD completion mirrored those seen in other regions of the world. Discussion between patient and their physician and patient’s functional impairment were the factors independently associated with completion of AD. Therefore, physicians need to be aware of the importance of discussions of AD with their patients.

## Background

Advance care planning for end of life is practiced in many parts of the world. It is a process that results in a written document, an advance directive (AD), which legally stipulates a person’s health care preference while they are competent to make decisions for themselves, and which is then used to guide decisions on life-sustaining treatment in the event that they become incapacitated.

The uptake of AD has been limited even in the countries where its use has existed for several years. The prevalence of AD in Australia was about 14% in one study, [1] and in a population in Canada, the prevalence of AD was 43.6% [2]. About a third of USA population have an AD [3]. At the hospital level, there is a low uptake in most branches of medicine other than oncology.

What is considered a good way to die differs from society to society [4]. The prevailing culture and the economic situation of a country determine the practice of end of life care in that country. Africa differs significantly from Western countries in terms of culture and economics. The factors that influence an individual’s uptake of AD are complex. They include patient, caregiver, legal, institutional, cultural, and religious factors, and while these factors may be similar between two societies, they do not have the same prevalence or contribute equally in each society.

In Western societies, where the factors that influence AD have been studied the most, the studies have been predominantly among elderly populations. Most of these studies have been prospective population-based surveys. Lovell *et al.,* in a systematic review, summarize factors that determine AD use amongst palliative care patients. It revealed that older age, college education, diagnosis of cancer, being white, previous illnesses, an individual’s knowledge and attitude, a health care provider’s knowledge and attitude, availability of hospice care, specialist palliative care treatment, and laws on AD were positively correlated with signing an AD. While suffering from dementia, being African American, having dependent children, avoidance of acknowledging death and dying, concerns about AD resulting in withdrawal of care, and lack of facilitative laws were among the factors hindering uptake of AD [5]. Overall, advanced age and terminal illness were the most common reasons for completing AD.

Advance directives are hardly completed in Africa and little has been done on this subject. The relevance of AD in an environment with limited health care facilities, as is the case in most countries in Africa, may itself be questionable. In such a setting, most patients do not have a true choice on end of life care since life-sustaining treatment may not be available. One can, however, still make the opposite argument that available health resources in these countries should be directed where they would have the most impact, and that one way of doing that would be to promote uptake of AD. A study addressing the question of relevance of AD amongst five focus groups in South Africa found that AD was considered relevant by all five focus groups [6]. There have also been concerted efforts led by WHO to develop palliative care in Africa to take care of the increasing number of terminally ill cancer and HIV patients [7, 8].

In most African societies, discussion of death and dying is considered a taboo. Yet, it is frowned upon when an individual takes unilateral decisions on issues of dying. The decisions on end of life care are preferably deferred to family members or community elders [9]. In Kenya, for example, 68.2% of respondents in one study indicated they would like a relative to be involved in end of life decision making [10]. The study was a population-based survey of public preferences and priorities for end of life care in Kenya done in Nairobi and Western Kenya. It found that the majority, 61.4%, preferred quality of life over quantity, i.e., extending life. One’s own home was the most commonly (51.1%) preferred place to die [10].

Kenya does not have a law on AD, and where practiced, it is usually under institutional policy. At our hospital, the policy on end of life care was enacted in 2012. It offers guidance on when end of life care discussions should be initiated. It also provides suggestions to health care providers on the categories of patients that should be considered for end of life care discussions. Our hospital has a 280-bed capacity. It is a private tertiary level hospital that provides specialty training in internal medicine, pediatrics, obstetrics and gynecology, surgery, anesthesiology, radiology, anatomic pathology, and clinical pathology. The Internal Medicine department has several subspecialties – oncology, cardiology, infectious diseases, pulmonology, gastroenterology, nephrology, critical care, dermatology, neurology, hematology, endocrinology, rheumatology, psychiatry, and palliative care. The palliative care unit has one specialist doctor who heads a team of allied professionals.

The objectives of our study were to determine the proportion of terminally ill patients with AD and to identify the factors that influence AD completion at our hospital.

## Methods

We conducted a retrospective survey using all available records of terminally ill patients seen at Aga Khan University Hospital, Nairobi, between July 2010 and December 2015, and that met the inclusion criteria of the study.

**Fig. 1:**
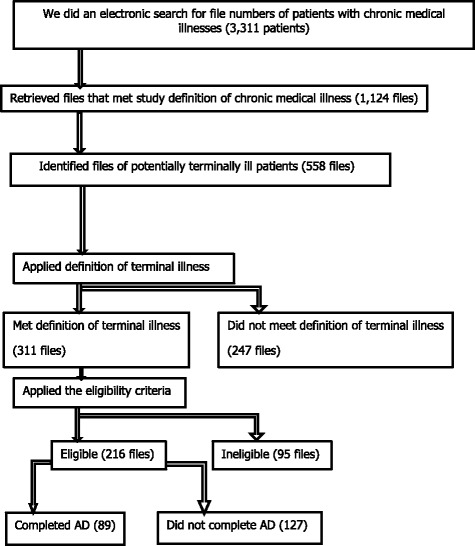
Recruitment Process

The process that was followed in identifying the files to include in the study is outlined in Fig. 1.

Terminal illness was defined as a disease that could not be cured or adequately treated and that was reasonably expected to result in the death of the patient within a short period, which was arbitrarily defined as within six months. A file that had records of a living will, a limitation of care document, a do-not-resuscitate order, or an appointment of surrogate by durable power of attorney for health care was considered to denote that the patient had completed AD.

We included only records of patients that were or above age 18 years and whose first and last consultations at our hospital were at least three months apart.

We excluded a record without a diagnosis, a record that was indeterminate for the definition of terminal illness, a record that had no dates of first and last consultations, or one that had more than 30% of the questionnaire variables missing.

### Data management and analysis

Stata version 14.1 was used to analyze the data. Chi square test was used to analyze the relationship between proportions of categorical variables in the subjects that completed AD and in those who did not complete AD. Fisher’s exact test was used to examine the same categorical variables to take care of the small sample size. The factors found to be statistically significant in bivariate analysis were then subjected to multivariate regression analysis.

## Results

**Fig. 2:**
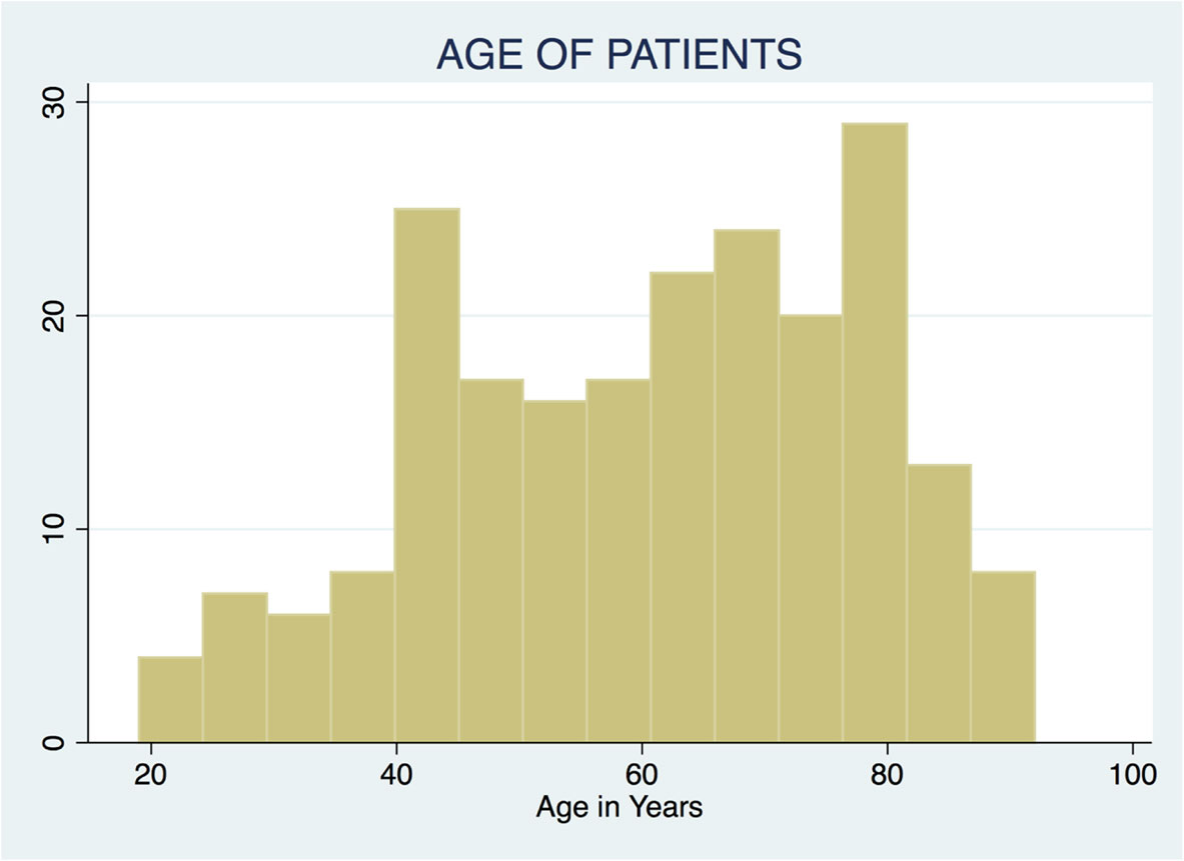
Histogram of age of patients

The numbers of records that were found in the study are depicted in Fig. 1. The patients’ characteristics are depicted in Table 1. The diagnoses of study subjects are depicted in Table 2. The mean age of study subjects was 60.45 years; the median age was 63 years. The minimum and maximum ages were 19 years and 93 years, respectively. Figure 2 depicts a histogram of the patients ages.

**Table 1:** Patients’ Characteristics

Patients Characteristics		
	n	%
Age Group		
< 45 years	57	22.53
45-65 years	88	34.78
> 65 years	108	42.69
Gender		
Male	113	52.31
Female	103	47.69
Nationality		
Kenyan	183	84.72
Non-Kenyan	83	15.28
Ethnicity		
African	151	69.91
Asian	48	22.22
Caucasian	13	6.02
Others	4	1.85
Marital Status		
Divorced	3	1.4
Married	164	76.64
Single	18	8.41
Widowed	29	13.55
Parental Status		
A Parent	189	87.5
Not a Parent	27	12.5
Education		
No formal Education	5	2.43
Primary	17	7.94
Secondary	25	11.68
Tertiary	167	78.04
Socio-economic class		
Semi-skilled	27	12.74
Unskilled	86	40.57
Highly skilled	99	46.7
Religion		
Hindu	20	9.35
Islam	37	17.29
Judaism	1	0.49
Protestant	140	65.42
Catholic	15	7.01
Others	1	0.47

**Table 2:** Diagnoses of subjects with and without AD

	Patient with AD	Patient without AD
Oncology		
Brain Cancer	1	2
Breast Cancer	16	16
Esophageal Cancer	2	3
Cervical Cancer	5	6
Cholangiocarcinoma	3	8
Colorectal Carcinoma	8	6
Endometrial Cancer	2	1
Gastric Cancer	8	1
Hepatocellular Carcinoma	9	10
Lung Cancer	8	6
Nasopharyngeal Carcinoma	2	4
Neuroendocrine Cancer	2	1
Ovarian Cancer	2	0
Pancreatic Cancer	4	1
Prostate Cancer	3	8
Non-Oncology		
Advanced HIV	0	6
End Stage Renal Disease	3	19
Advanced Heart Failure	2	16
Advanced Dementia	2	2
Advanced Interstitial Lung Disease	3	4
Advanced COPD	2	2
Advanced Liver Cirrhosis	2	5

**Fig. 3:**
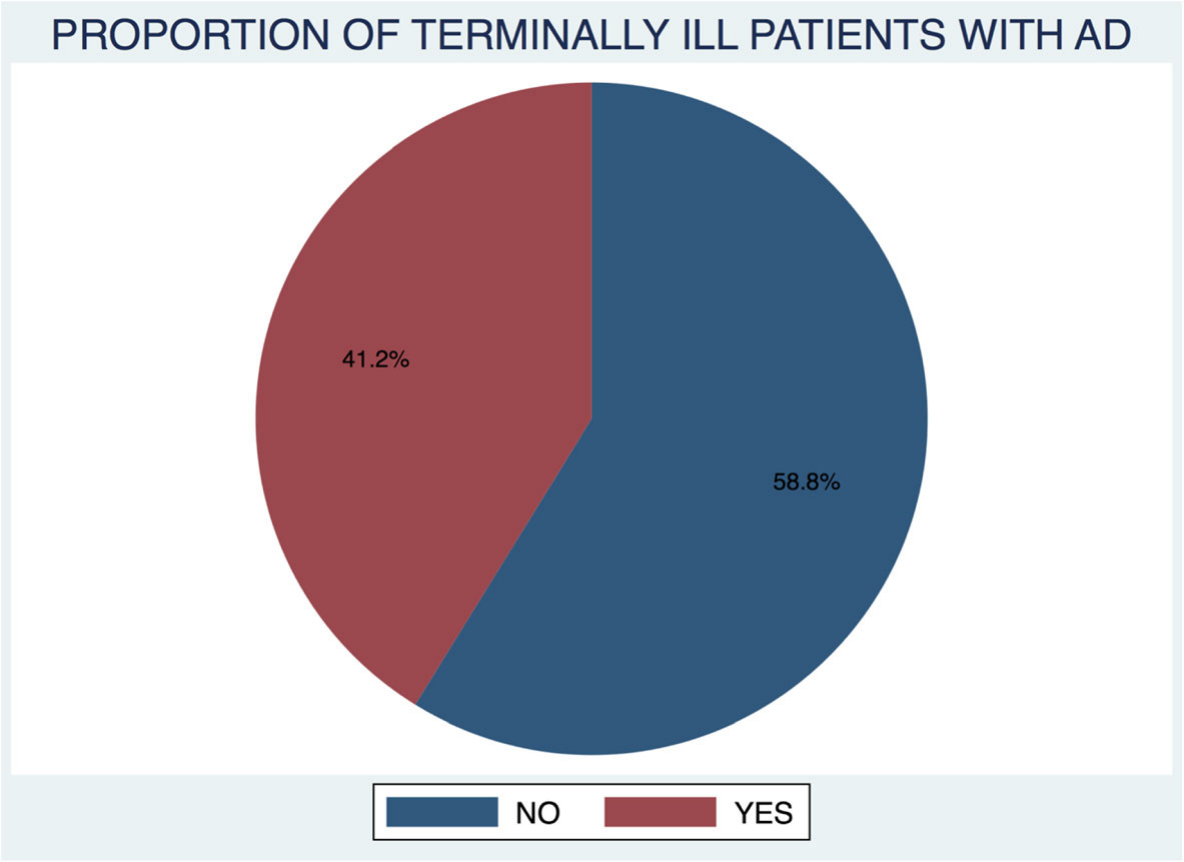
proportion of terminally ill patients with AD

In total, 216 records of terminally ill patients met the inclusion criteria: 89 were of patients that had advance directives and 127 were of those that did not have advance directives. These proportions are depicted in Fig. 3.

**Fig. 4:**
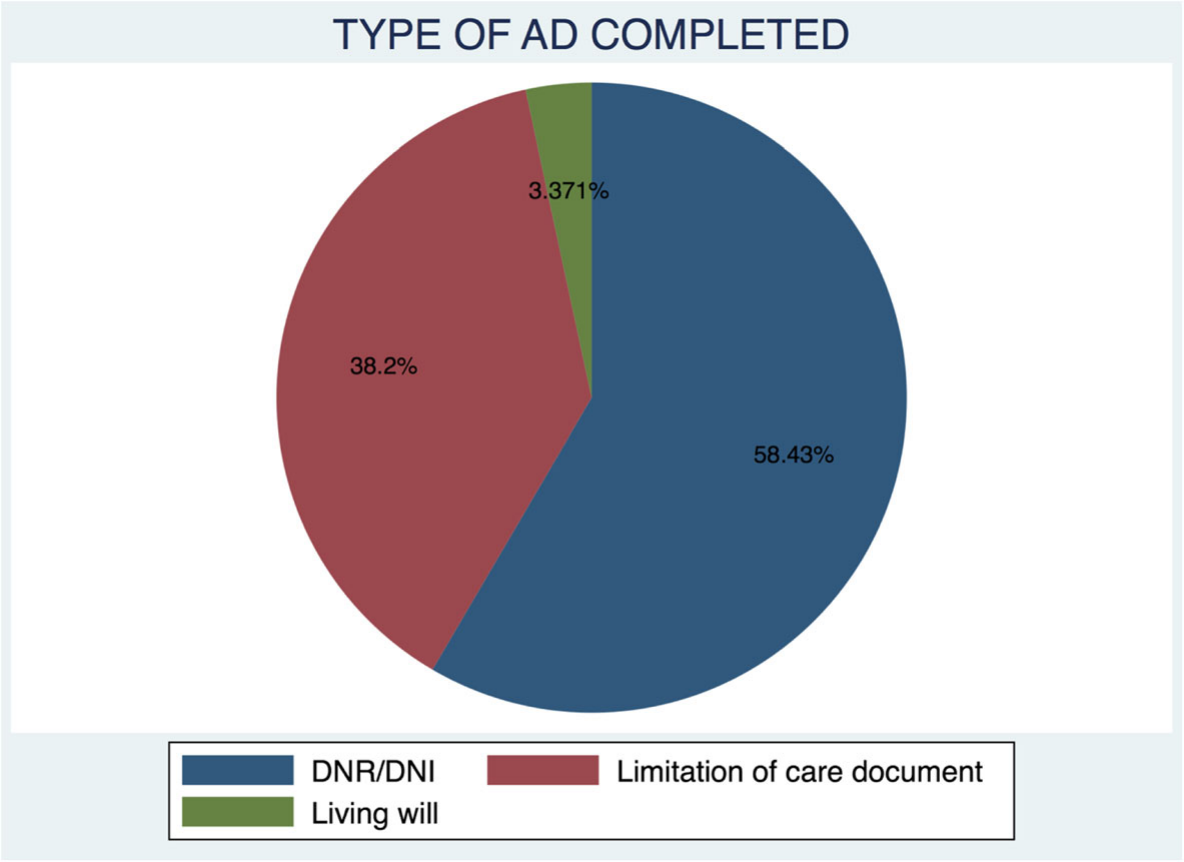
Types of AD Completed

The most common type of advance directives was a limitation of care document at 58.3%. Figure 4 depicts the types of AD that were completed by patients.

The proportion of terminally ill patients that had completed AD was 41.2%. The factors that were associated with completion of AD on bivariate analysis were history of ICU admission, history of endotracheal intubation, the functional status of the patient, the medical specialty taking care of the patient, a caregiver discussing the AD with the patient, and a palliative care specialist reviewing the patient. The results of bivariate analysis of the factors associated with AD completion are depicted in Table 3.

**Table 3:** Comparison of subjects with and without AD

	*n* =216	Patients without AD (*n* = 89)		Patients without AD (*n* = 127)		
	%	*n*	%	*n*	%	*P* Value
Age Group						0.921
< 45 years	21.3	19	21.35	27	21.26	
45-65 years	35.19	30	33.71	46	36.22	
> 65 years	43.52	40	44.94	54	45.52	
Gender						0.903
Female	47.69	42	47.19	61	48.73	
Male	52.31	47	52.81	66	51.97	
Ethnicity						0.377
African	69.91	67	75.28	84	66.14	
Asian	22.22	18	20.22	30	23.62	
Caucasian	6.02	3	3.37	10	7.87	
Others	1.85	1	1.12	3	2.36	
Marital Status						0.313
Divorced	1.4	1	1.12	2	1.6	
Married	76.64	67	75.28	97	77.6	
Single	8.41	11	12.36	7	5.6	
Widowed	13.55	10	11.24	19	15.2	
Parental Status						0.715
A Parent	87.5	77	86.52	112	88.19	
Not a Parent	12.5	12	13.48	15	11.81	
Number of Children						0.779
No Child	12.98	12	13.95	15	12.3	
1-5 Children	64.42	53	61.63	81	66.39	
≥ 5 Children	22.6	21	24.43	26	21.31	
Education						0.221
No formal Education	2.43	4	4.49	1	0.8	
Primary	7.94	9	10.11	8	6.4	
secondary	11.68	9	10.11	16	12.8	
Tertiary	78.04	67	75.28	100	80	
Socio-economic class						0.889
Unskilled	40.57	38	42.7	48	39.02	
Semi-skilled	12.74	11	12.36	16	13.01	
highly skilled	46.7	40	44.94	59	47.97	
Religion						0.189
Hindu	9.35	4	4.55	16	12.7	
Islam	17.29	18	20.45	19	15.08	
Judaism	0.49	0	0	1	0.79	
Protestant	65.42	59	67.05	81	64.29	
Catholic	7.01	6	6.82	9	7.14	
Others	0.47	1	1.14	0	0	
Insurance Status						0.295
Has Health Insurance	61.35	57	65.52	70	58.33	
No Health Insurance	38.65	30	34.48	50	41.67	
AD Discussion						<0.05
Caregiver Discussed AD	48.15	82	92.13	22	17.32	
Caregiver didn’t Discuss AD	51.85	7	7.87	105	82.68	
No. of Morbidities						0.25
1 - 3 Morbidities		47	52.81	51	40.16	
> 3 Morbidities	38.43	28	31.46	55	43.31	
Number of Admission						0.43
1 - 3 Admissions	93.98	85	95.51	118	92.91	
> 3 Admissions	6.02	4	4.49	9	7.09	
Specialty						<0.05
Oncology	68.52	75	84.27	73	57.48	
Non-Oncology	31.48	14	15.73	54	42.52	
History of Surgery						0.201
History of Major Surgery	9.14	4	4.49	11	9.09	
No History of Major Surgery	92.86	85	9551	110	90.91	
ICU Admission						<0.05
Previous ICU Admission	40.47	20	22.47	69	53.17	
No Previous ICU Admission	59.53	67	77.53	59	46.83	
Intubation History						<0.05
Previous Intubation	34.42	17	19.1	57	45.24	
No Previous Intubation	65.52	72	80.9	69	54.76	
Palliative Care Consult						<0.05
Palliative Care Consult	54.88	71	80.68	47	37.01	
No Palliative Care Consult	45.12	17	19.88	80	62.99	
Functional Status						<0.05
Functional Impairment	83.33	82	92.13	98	77.17	
No Functional Impairment	16.67	7	7.87	29	22.83	
Household Composition						0.186
Lives alone	7.87	10	11.24	7	5.51	
Lives with Immediate Family	64.35	52	58.43	87	68.5	
Lives with Extended Family	27.78	27	30.34	33	25.58	

On multivariate regression analysis, the discussion of AD with a caregiver and the patient’s functional impairment were the only factors with statistically significant association with completion of AD. The results of the multivariate analysis are depicted in Table 4.

**Table 4:** Multivariate analysis of factors associated with AD completion

	RRR^a^	*P* Value	[95% Confidence Interval]	
Caregiver discussed AD	40.56891	<0.05	15.05035	109.3554
Previous ICU^b^ Admission	0.6650326	0.582	0.1558932	2.836996
Intubation history	0.4509578	0.304	0.0987241	2.059912
Palliative care consult	1.43966	0.474	0.5305328	3.906678
Functional Impairment	4.169589	0.021	1.243331	13.98298
Specialty	1.010565	0.986	0.3145422	3.246757

^a^RRR – Relative Risk Ratio

^b^ICU – Intensive Care Unit

Sixty (67%) the of subjects who had completed AD had their wishes honored by the attending doctors.

## Discussion

This study was a retrospective survey to determine the proportion of terminally ill patients that had completed AD and to determine the factors that were associated with AD completion.

We found that 41.2% of terminally ill patients had completed AD. Sittisombut *et al.* found even a higher completion rate (80%) amongst terminally ill patients in Thailand [11]. Both our study and Sittisombut’s looked at terminally ill patients, and this could explain why our studies had proportionally higher completion rate of AD compared to, for example, Mezey *et al.’*s study, in which only 20.4% had completed AD but which included both terminally ill and non-terminally ill patients [12]. The rate of AD completion in our study was not very high when compared to the rates of AD completion reported in the West. It can certainly be improved with better participation of the primary physicians.

Limitation of care documents and DNR orders were the most common type of AD completed in our study. There were only three living will documents completed. There was no single power of attorney by proxy documentation filled. Our findings contrast with those of the Alano *et al.* study where up to 85% of subjects had completed a living will [13]. The lower proportion of living will documents found in our study could be attributed to the lack of AD laws in our country, but cultural factors may also have played a role.

There was no significant association between ethnicity and completion of AD in our study. In the USA, African-Americans and other minority ethnic groups were less likely to have AD compared to the Caucasians [14]. In our study the majority of patients were African (69.92%) followed by Asians (22.22%). Caucasians constituted only 6.02% of patients. Completion of AD is known to be generally higher amongst Caucasians, and they were under-represented in our study. In addition, a pertinent point to note is that our hospital is mostly patronized by well-educated, high and middle class clients, irrespective of their ethnicity. The homogenous socio-economic status of our patients could thus be the reason an association between ethnicity and AD completion was not seen in our study.

Many patients learn about advance directives for the first time from their doctor. Therefore, promoting physicians’ understanding of AD is one way of promoting the uptake of AD [15]. Similar to the positive association between caregivers discussing AD with their patients and completion of AD that was found in this study, Walker *et al.* found that after controlling for demographic and clinical confounders, physicians had the most important role in determining uptake of AD. Palliative care includes advance care planning as one of the services offered, and it is expected, as was seen in this study, that a palliative care specialist consultation would be associated with a higher completion rate of AD. Therefore, health institutional policies and protocols that ensure attending physicians discuss AD with all terminally ill patients have great potential to improve completion rates.

Moody *et al.* found functional impairment to be correlated with completion of AD [16]. We similarly found that the degree of functional impairment as measured by the Karnofsky performance scale index was positively associated with completion of AD. In interview-based studies in the United states, when patients were asked what factors influenced them to complete AD, functional impairment, loss of independence, previous experiences with critical care, or experiencing a friend or relative undergo a prolonged admission in critical care were some of the factors mentioned [12, 17]. In our study, we found a similar association between completion of AD and history of critical care admission or endotracheal intubation. We did not, however, find an association between completion of AD and the number of comorbidities, the number of hospital admissions, or the history of major surgery, all of which have been found to be positively correlated with AD completion in other studies.

Lovell *et al.,* in a systematic review of factors that influence AD completion amongst palliative care patients, reported that history of malignancy was positively correlated with completion of AD. They also reported that diagnoses of chronic obstructive pulmonary disease (COPD) and dementia were negatively correlated with completion of AD [5]. Butler *el al* found that most heart failure patients did not have AD documented in their files [18]. In our study, there was no independent association found between a diagnosis of malignancy and AD completion, but as was found in the Butler *et al.* study, there was a negative association between a heart disease diagnosis and completion of AD.

In our study, only six (2.78%) patients had HIV, and none of them had completed AD. All of them had well-controlled disease. Barocas *et al.* studied AD completion amongst people living with HIV and found that only a quarter of people living with HIV had completed AD. The factors associated with AD completion in their study were diagnosis of AIDS, neurological, cardiovascular, chronic kidney disease, or malignancy. They made the point that since HIV had become a chronic disease and patients were more likely to die from other comorbid conditions, opportunities were being missed to complete AD in this population.

The mean age in our study was 60.45 years. There was no association of age with completion of AD in our study. The mean age in Sittisombut *et al.* study was 57.5 years. The mean age in Mezey *et al.* study was 52 years. In the Sittisombut *et al.* study, most subjects were above age 60 years. They did not, however, report the association of age with AD completion. In the studies done in the Western countries, advanced age is an important determinant of AD completion [2, 19]. These studies were however not among terminally ill patients. It is possible that a diagnosis of terminal illness eliminates the influence of advanced age in AD completion.

We did not find an association between parental status and AD completion. Neither was there an association between the number of children a subject had and completion of AD. Nilsson *et al.* found a positive correlation between having dependent children and completion of AD [20]. Most of our subjects were married, but there was no association between marital status and AD completion. It is not clear why family dynamics as reflected by the factors mentioned here had no association with AD completion. It could be because of the small sample size. For example, very few subjects in our study lived alone.

One’s occupation type or whether they had medical insurance did not have an association with completion of AD in our study. One of the barriers to completion of AD found among the African-Americans was lower socioeconomic status [21]. In a hospital based study, Mezey *et al.* found a positive correlation between one’s salary and one’s health insurance status with completion of AD [12]. The association between AD completion and cost may not have been found in our study because it was done in a tertiary private hospital where most patients had health insurance.

Most of the subjects in our study were non-Catholic Christians (65.42%) or Muslims (17.29%). There was, however, no significant association found between religion and completion of AD.

The proportions of males and females in our study were 52.31% and 47.69%, respectively. Perkin’s *et al.* addressed the role of gender in completion of AD. They found that men preferred death over futile care, and that men addressed only the functional aspects of end of life care. This contrasted with women who addressed other aspects as well [22]. In our study, there was no significant difference between proportions of males and females who completed AD.

The majority of patients in our study had obtained tertiary level education (78.04%). The proportion of patients with tertiary education was higher in the people who did not complete advance directives (80%) compared to those who had completed (75.28%). However, education level attained did not have a statistically significant association with completion of AD in our study. Mezey *et al.* found education to be the most important factor in determining uptake of AD [12]. In another hospital-based study in Thailand, Sittisombut *et al.* the majority of patients had attained grade 4–6 level of education, with only about one third having attained above grade 9 level education. In their study, as was in ours, there was no correlation between education and completion of AD [11]. One reason why our results are similar to Sittisombut’s could be the fact that in both studies the decisions to complete AD were made in most cases by the patient after discussions with their family, as opposed to an individualistic approach to AD completion.

There are patient factors associated with AD such as knowledge and attitude of patients that we could not study due to the retrospective nature of our study. There are also aspects of patient-physician relationship important for AD completion that we could not study. The role played by lack of laws on AD in Kenya could also not be studied. The influence of patients’ relatives in influencing AD could also not be studied.

Due to the retrospective nature of our study, we could not focus on the role of religion and spirituality, patients’ preexisting knowledge about advanced directives, patient-physician relationship, and influence of the relatives on decision making. However, all these factors are known to play a role in AD completion [23] [24].

Despite these limitations, our study shows that the factors associated with AD in Kenya are not different from those reported in the studies done in Western countries. It also re-demonstates the important role played by physicians in promoting AD use. Despite reporting from a developing country, our study demonstrates good uptake of AD in our hospital amongst terminally ill patients.

## Conclusion

The proportion of terminally ill patients that had AD in their medical records was significant, however, most terminally ill patients did not have AD. Most of the factors associated with AD completion were the same as those seen in other regions of the world. Among them, discussion between a patient and their physician and functional impairment of the patient were the factors independently associated with completion of AD. We conclude that physician involvement and early discussion with the patients are key components associated with AD completion. Similar but larger studies, done prospectively and incorporating patients from public hospitals, could identify other factors that could be improved to enhance completions of AD in our population. However, some factors known to influence AD completion in the United States of America and Western European countries such as age did not have a correlation with AD in our study, perhaps reflecting a relatively younger population in our setup.

To improve uptake of AD in our setting, we should encourage more physician - patient discussion as this was the main modifiable influence of AD uptake in our setting.
